# A double-blind, randomized, multicenter phase 2 study of prasugrel versus placebo in adult patients with sickle cell disease

**DOI:** 10.1186/1756-8722-6-17

**Published:** 2013-02-17

**Authors:** Ted Wun, Denis Soulieres, Andrew L Frelinger, Lakshmanan Krishnamurti, Enrico M Novelli, Abdullah Kutlar, Kenneth I Ataga, Charles L Knupp, Lillian E McMahon, John J Strouse, Chunmei Zhou, Lori E Heath, Chuke E Nwachuku, Joseph A Jakubowski, Jeffrey S Riesmeyer, Kenneth J Winters

**Affiliations:** 1 University of California, Davis Cancer Center, 4501 X St., Ste. 3016, Sacramento, CA, 95817, USA; 2 CHUM-Notre-Dame, Montreal, QC, Canada; 3 Children’s Hospital Boston, Harvard Medical School, Boston, MA, USA; 4 Children’s Hospital of Pittsburgh, Pittsburgh, PA, USA; 5 University of Pittsburg Medical Center, Pittsburg, PA, USA; 6 Medical College of Georgia, Augusta, GA, USA; 7 University of North Carolina at Chapel Hill, Chapel Hill, NC, USA; 8 ECU-Brody School of Medicine, Greenville, NC, USA; 9 Boston Medical Center, Boston, MA, USA; 10 The Johns Hopkins University School of Medicine, Baltimore, MD, USA; 11 Eli Lilly and Company, Indianapolis, IN, USA; 12 Daiichi Sankyo, Inc, Edison, NJ, USA

**Keywords:** Prasugrel, Sickle cell disease, Thienopyridine, Platelet function

## Abstract

**Background:**

Platelet activation has been implicated in the pathogenesis of sickle cell disease (SCD) suggesting antiplatelet agents may be therapeutic. To evaluate the safety of prasugrel, a thienopyridine antiplatelet agent, in adult patients with SCD, we conducted a double-blind, randomized, placebo-controlled study.

**Methods:**

The primary endpoint, safety, was measured by hemorrhagic events requiring medical intervention. Patients were randomized to prasugrel 5 mg daily (n = 41) or placebo (n = 21) for 30 days. Platelet function by VerifyNow® P2Y12 and vasodilator-stimulated phosphoprotein assays at days 10 and 30 were significantly inhibited in prasugrel- compared with placebo-treated SCD patients.

**Results:**

There were no hemorrhagic events requiring medical intervention in either study arm. Mean pain rate (percentage of days with pain) and intensity in the prasugrel arm were decreased compared with placebo. However, these decreases did not reach statistical significance. Platelet surface P-selectin and plasma soluble P-selectin, biomarkers of in vivo platelet activation, were significantly reduced in SCD patients receiving prasugrel compared with placebo. In sum, prasugrel was well tolerated and not associated with serious hemorrhagic events.

**Conclusions:**

Despite the small size and short duration of this study, there was a decrease in platelet activation biomarkers and a trend toward decreased pain.

## Background

Sickle cell disease (SCD) (the common term used for the clinical syndrome that results from homozygous hemoglobin S; compound heterozygous Hb S/C; and compound heterozygous Hb S/β^0/+^-thalassemia) results from a mutation in the β-globin gene. The clinical manifestations of disease are due to hemolysis, and intermittent microvascular occlusion marked by painful vaso-occlusive crisis (VOC) and eventual end-organ damage from repeated bouts of ischemia-reperfusion injury resulting in significant disabilities and early mortality. SCD pathophysiology is multi-factorial. The reduced intracellular solubility of HbS leads to intra-erythrocytic hemoglobin polymerization and loss of red cell deformability under conditions of hypoxia and acidosis [1]. The sickle red blood cell is also abnormally adhesive to activated endothelial cells even in the absence of sickling [2]. Neutrophils and monocytes are activated and contribute to vascular occlusion [3-6]. Numerous studies have also shown activation of the hemostatic system, including the coagulation cascade and platelets [7-9]. Activation of all these cells and the coagulation system likely contributes to a prothrombotic state producing the acute and chronic disease manifestations.

Previous work, using various soluble and immunological biomarkers, has shown that platelets are activated in patients with SCD [10-12]. Some data have suggested increased platelet activation during VOC [12-14]. Possible mechanisms whereby activated platelets contribute to the pathogenesis of VOC include release of thrombospondin 1 during platelet activation that supports aberrant red blood cell (RBC) adhesion to endothelium and matrix proteins, and platelets directly mediating RBC adherence to endothelial cells and capture onto neutrophils and monocytes (heterotypic cell-cell adherence; and activation of neutrophils and monocytes via formation of platelet-monocyte and platelet-neutrophil aggregates). The activated leukocytes augment the overall inflammatory state and promote further vascular and tissue damage.

There have been studies of antiplatelet agents for SCD patients including use of aspirin [15] and the first generation thienopyridine adenosine diphosphate (ADP) receptor inhibitor, ticlopidine [16,17]. In general, these studies have been relatively small, underpowered, and without control groups. Prasugrel, a third-generation platelet P2Y12 ADP receptor antagonist, is FDA-approved in combination with aspirin for treatment of patients with acute coronary syndrome undergoing percutaneous coronary intervention. We performed a phase 2, double-blind, randomized trial of prasugrel compared with placebo in patients with SCD. The primary safety outcome was treatment-emergent hemorrhagic events requiring medical intervention. Secondary safety outcomes included all adverse events (AEs) including those that required study drug discontinuation. The efficacy outcome measures were frequency and intensity of pain ascertained by self-administered pain diary; frequency of pain requiring medical attention; pharmacodynamic effects on platelets measured by VerifyNow® P2Y12 reaction units (PRU) and vasodilator-associated stimulated phosphoprotein (VASP) platelet reactivity index (PRI); and biomarkers of platelet activation. We hypothesized that prasugrel would: 1) be well tolerated, 2) inhibit platelet activation, and 3) decrease pain.

## Methods

This was a double-blind, randomized, multicenter trial to assess the safety of prasugrel 5 mg PO (by mouth) daily compared with placebo in adult patients with SCD (clinicaltrials.gov #NCT01167023). The study was conducted at 18 sites in the United States and Canada from 26 August 2010 to 13 June 2011. The study was approved by the local institutional review boards and was performed in compliance with principles of good clinical practice (GCP) and in accordance with the provisions of the Declaration of Helsinki. Each patient voluntarily signed an informed consent document before entering the study.

### Eligibility

To be included in the study, patients had to be adults 18 to 55 years of age with SCD (genotypes HbSS, HbSC, HbS-β^0^-thalassemia and HbS-β^+^-thalassemia), who did not have a diagnosis of acute VOC within 30 days of the study screening visit. Patients with severe hepatic or renal dysfunction, hematocrit <18%, and those at risk of excessive bleeding complications including platelet count <100,000 per cubic millimeter, prior history of bleeding disorders, hemorrhagic or ischemic stroke, retinal hemorrhage, a transient ischemic attack (TIA), or intracranial hemorrhage were excluded from the study. We felt these conservative exclusion criteria were warranted given this new patient population. Use of aspirin or other antithrombotics was not allowed within 10 days of entry or during the study. Non-steroidal anti-inflammatory drugs (NSAIDs) for treatment of pain were not permitted in the 5 days prior to randomization or for ≥5 consecutive days during the study period. Use of hydroxyurea was permitted in patients already on a stable dose 30 days prior to randomization.

### Study design

**Figure 1:**
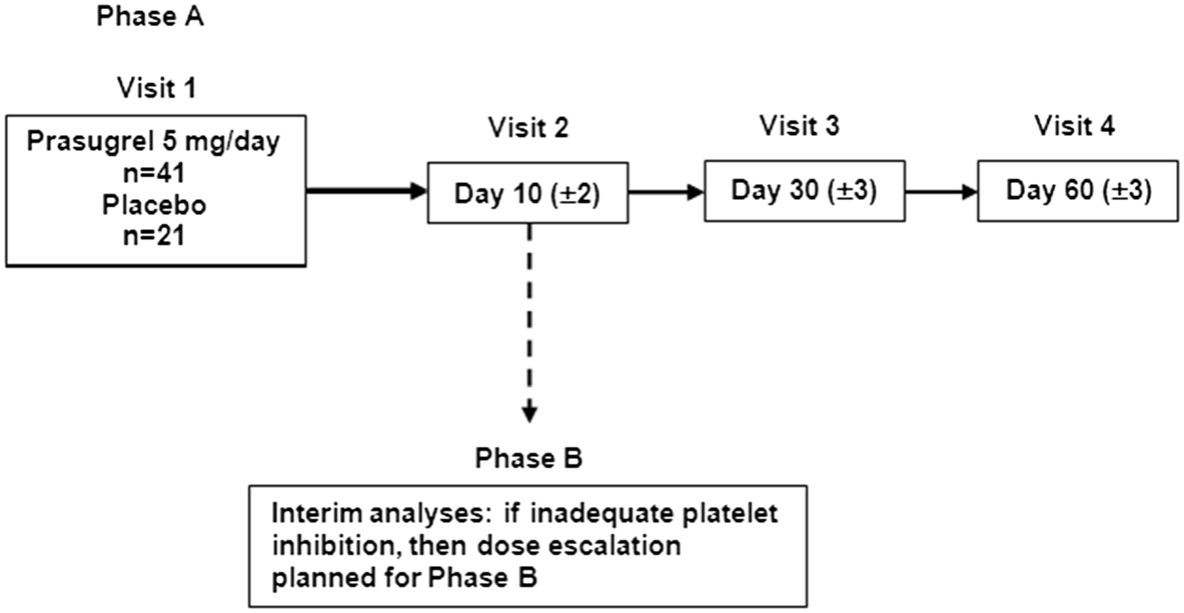
**Adaptive study design Phase A and Phase B.** Decisions about dose allocations were made as the trial progressed. If interim analysis of pharmacodynamic data revealed insufficient platelet inhibition in the first 16 patients randomized to 5-mg daily prasugrel, the dose was to be escalated to 7.5 mg. Dotted line denotes dose escalation plan per protocol; no dose escalation occurred occur during the study

The study consisted of 2 phases (Phase A and Phase B) (Figure 1) and employed an adaptive design with decisions about dose allocations made as the trial progressed. During each phase, patients were randomized to prasugrel or placebo in a 2:1 manner and stratified by sickle-cell genotype (HbSS, HbS-β^+^-thalassemia and HbS-β^0^-thalassemia genotype patients in one stratum, and HbSC genotype patients in another stratum). We stratified in this way because some data [18,19], though not all [10,14], suggest more pronounced platelet activation in patients with HbSS than HbSC. In addition, our limited studies showed no difference in platelet activation between Hb S-β^+^-thalassemia and HbSS patients [10]. Patients underwent screening (Visit 0) and returned to the site for Visit 1 (0 to 10 days after screening), Visit 2 (10 ± 2 days after Visit 1), and Visit 3 (30 ± 3 days after Visit 1). Patients were contacted by phone for Visit 4 (30 days after last dose of study drug) to collect information on adverse events (AEs) and serious adverse events (SAEs).

All patients randomized to prasugrel during Phase A received a 5-mg/day dose. When pharmacodynamic (PD) data from Visit 2 were available for ≥16 patients treated with prasugrel, a Data Monitoring Committee (DMC) assessed the results and reviewed initial safety data prior to making a determination regarding the Phase B prasugrel dose. If at Visit 2, ≥ 60% of ≥ 16 patients treated with prasugrel in Phase A had the equivalent of < 25% inhibition of platelet aggregation (IPA) as measured by light transmission aggregometry (LTA), then a 7.5-mg/day prasugrel dose was to be used in Phase B for patients who weighed ≥ 60 kg, and a 5-mg/day prasugrel dose would be used in patients who weighed < 60 kg. Otherwise, Phase B was to use a5-mg/day prasugrel dose for all patients, regardless of weight. After the DMC determined the Phase B dose, future patients were randomized into Phase B, while all patients randomized in Phase A completed their scheduled follow-up. Based on the accumulated data, as reviewed by the DMC, a 5-mg daily dose was used for the remainder of the study. The duration of active treatment and pain assessment was 30 days and the total duration with follow-up was 60 days.

### Endpoint assessment

The primary endpoint was the incidence of hemorrhagic events requiring medical attention: an unscheduled visit or call to a medical provider due to a complaint of bleeding while on study drug. Bleeding events that did not require medical attention were captured, in retrospect, as adverse events at routine assessments. This endpoint was chosen because sickle cell patients represented a new population for this drug and it was felt necessary to have robust safety data in adults prior to proceeding with a planned study in pediatric patients. Endpoint ascertainment was achieved by interviewing patients at each visit. Additional safety evaluations included the assessment of clinical laboratory test results, vital sign measurements, 12-lead electrocardiogram results, fundoscopies, and AEs. Information on SAEs was collected at occurrence as well as by telephone interview 30 days after the last dose of study drug. Disease-related treatment emergent adverse effects (TEAEs) including VOC, acute chest syndrome, hepatic sequestration, and stroke were reported as AEs or SAEs as appropriate.

Efficacy was a secondary endpoint as assessed by incidence of any pain events requiring medical attention in the study. In addition, the efficacy of prasugrel (5 mg) compared with placebo was measured by monitoring the frequency and intensity of pain related to SCD as recorded in patient pain diaries each day for 30 days. A scale of 0 to 9 was used to evaluate pain intensity, with 0 indicating no pain, and 9 indicating unbearable pain.

### Pharmacodynamic and biomarker evaluations

Platelet inhibition was assessed by the Accumetrics VerifyNow® P2Y12 assay and the VASP phosphorylation assay as previously described [20,21]. Assay data was reported as P2Y12 reaction units (PRU) and percent inhibition (device reported) for the VerifyNow® P2Y12 assay and platelet reactivity index (PRI) for VASP.

Platelet activation was assessed by immunoassay determination of plasma, serum, and cellular biomarkers of platelet activation. Multi-color fluorescent activated cell sorting (FACS) and monoclonal antibodies were used to determine platelet P-selectin expression using previously published protocols [22,23]. Soluble P-selectin (sP-selectin), soluble CD40 ligand (sCD40L), thromboxane B2 (serum TXB2), and prothrombin fragment F1.2 (F1.2) were determined using standard enzyme-linked immunoassays according to manufacturer’s directions.

Pharmacodynamic parameters and biomarkers were assessed at Visits 1, 2, and 3. VerifyNow® P2Y12 was performed locally; platelet surface P-selectin was determined at the Center for Platelet Research Studies; and all other evaluations were performed at a central laboratory. Approximately 30–50 mL of venous blood was collected by venipuncture at each visit for use in PD and biomarker testing.

### Statistical analysis

Patient characteristics were summarized by treatment group with values presented as mean ± standard deviation (SD) for continuous variables, and counts (percentages) for categorical variables. An analysis of variance (ANOVA) model for continuous variables and a Fisher’s exact test for the categorical variables were used for comparisons between treatment groups. The number and percentage of patients experiencing safety endpoints including the frequency of hemorrhagic events requiring medical intervention were summarized by treatment group.

Pain rate and pain intensity were summarized by treatment group using descriptive statistics. Pain rate was defined as the number of days reported with any pain related to SCD divided by the number of daily pain diaries completed. Pain intensity was the sum of a patient’s pain scores on a scale of 0 to 9 divided by the number of daily pain diaries completed. An analysis of covariance (ANCOVA) model with baseline pain intensity, treatment group, and sickle-cell genotype (HbSS, HbS-β^0^-thalassemia and HbS-β^+^-thalassemia in one stratum and HbSC in another stratum) in the model was used for treatment group comparison. A sensitivity analysis using only data from patients who completed 20 or more daily pain diaries was performed to verify that missing data did not introduce bias. The number of patients experiencing a pain event that required medical attention was compared between treatment groups using Fisher’s exact test.

Pharmacodynamic and biomarker results were summarized by treatment group and visit. The post-baseline results for each PD and biomarker parameter were analyzed using a mixed-effects model repeated measures (MMRM) analysis with the fixed effects of treatment, baseline parameter result, sickle-cell genotype, visit, and visit-by-treatment interaction, as well as a patient random effect included in the model. A compound symmetry variance-covariance structure was used to estimate within-patient errors.

Because a more conventional way to stratify analysis for sickle cell genotypes is to compare HbSS and HbS-β^0^-thalassemia with HbSC and HbS-β^+^-thalassemia (based on disease severity), the ANCOVA analysis for pain rate and pain intensity and the MMRM analysis for PD and biomarker parameters were repeated with adjustment of genotype based on the conventional stratification. This sensitivity analysis did not change any of the results for statistical significance on the treatment comparison.

The sample size calculation for this study is limited due to the rarity of the primary endpoint of hemorrhagic events requiring medical intervention, and so is supported by a power calculation for the efficacy endpoints. A sample size of 60 patients with the ratio of prasugrel to placebo as 2:1 provides 80% power to detect approximately 60% reduction in the mean pain rate at the 2-sided 0.05 level. This results in the ability to detect an effect size of 0.78.

## Results

**Figure 2:**
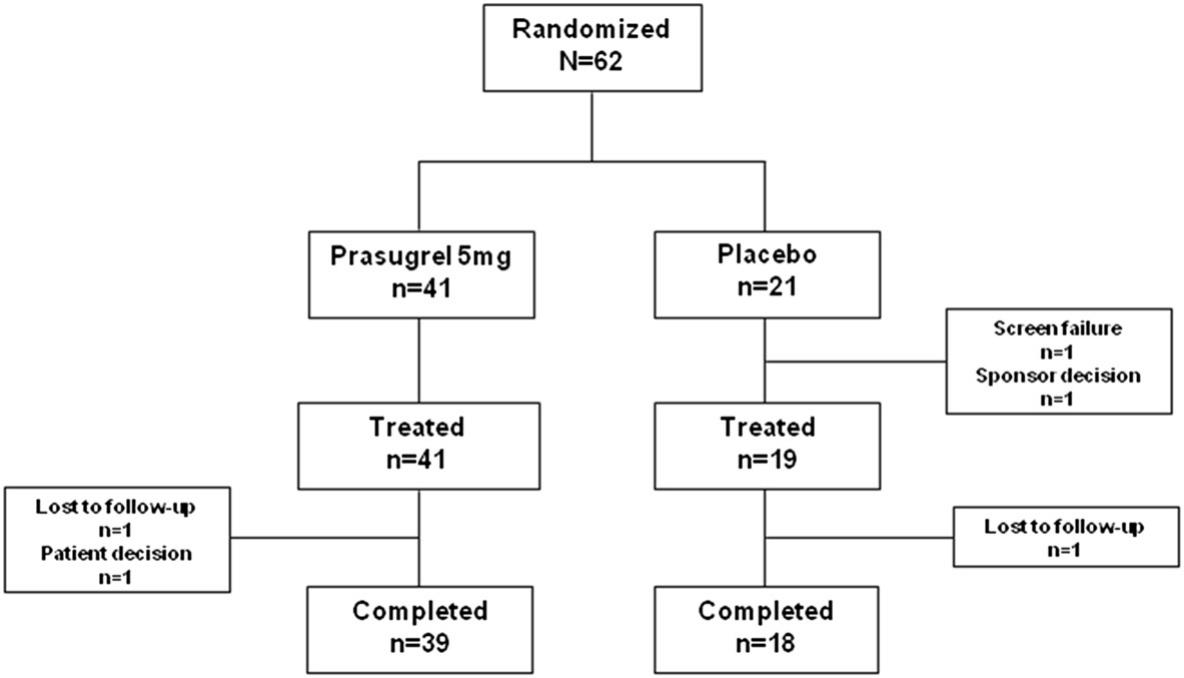
**Patient distribution.** A total of 62 patients were randomly assigned to treatment (prasugrel [41], placebo [21]) and were included in the Intent-to-Treat (ITT) analysis set

A total of 62 patients were randomly assigned to treatment (prasugrel [n = 41], placebo [n = 21]) and were included in the Intent-to-Treat (ITT) analysis set (Figure 2). A total of 57 patients completed the study (39 [95.1%] prasugrel; 18 [85.7%] placebo). No patients received aspirin, nor NSAIDs, during the study. One patient was found not to have SCD (β-thalassemia trait only) and another patient was enrolled and randomized as the study ended and it was decided not to proceed with treatment of that patient.

The baseline characteristics of the treatment groups were well balanced (Table 1). The mean pain intensity for all patients was low, likely reflecting that these patients were clinically stable without recent painful episodes. A greater proportion of patients randomized to prasugrel had a history of acute chest syndrome and systemic and pulmonary hypertension.

**Table 1:** Baseline characteristics and medical history

	Prasugrel 5 mg n = 41*	**Placebo n = 21**	**p value**
**Age (mean, years)**	32.9	31.5	0.553
**Female, n (%)**	21 (51.2)	9 (42.9)	0.598
**Body Weight (mean, kg)**	78.3	68.6	0.021
**Hydroxyurea**	18	9	
**Sickle-cell Genotype, n (%)**			>0.999
**Hb S/ β0 thalassemia**	2 (5.0)	1 (4.8)	
**Hb S/ β**$\tilde{+}$**thalassemia**	4 (10.0)	2 (9.5)	
**Hb SC**	10 (25.0)	5 (23.8)	
**Hb SS**	24 (60.0)	13 (61.9)	
**Pain Intensity**			0.473
**Mean**	1.8	2.4	
**Minimum**	0.0	0.0	
**Median**	0.0	2.0	
**Maximum**	9.0	7.0	
**Medical History, n (%)**			
**Vaso-occlusive crisis**	25 (61.0)	12 (57.1)	
**Acute chest syndrome**	9 (22.0)	2 (9.5)	
**Hepatic sequestration**	0	1 (4.8)	
**Splenectomy**	7 (17.1)	5 (23.8)	
**Systemic hypertension**	11 (26.8)	2 (9.5)	
**Pulmonary hypertension**	7 (17.1)	2 (9.5)	
**Renal failure**	1 (2.4)	0	
**Hepatic disease**^**†**^	1 (2.4)	0	
**Renal insufficiency**	0	1 (4.8)	
**Laboratory Values, mean (SD)**			
**Hemoglobin (g/L)**	10.4 (1.8)	9.8 (2.0)	0.297
**White Blood Cells (x10**^**9**^**/L)**	8.2 (3.4)	8.3 (2.7)	0.929
**Platelets (x10**^**9**^**/L)**	310.6 (180.4)	340.5 (117.5)	0.561

*1 patient was found not to have SCD but to have only β-thalassemia trait.

^†^Other than sequestration.

No patient experienced hemorrhagic adverse events that required acute medical intervention (Table 2), the primary endpoint of the study. Eight patients in the prasugrel arm experienced 9 hemorrhagic AEs, the majority of which were mild and possibly related to the study drug (Table 3) but did not result in study drug discontinuation. The proportion of SAEs and all TEAEs was similar between the groups. Most SAEs were pain events requiring medical intervention. The rate of study drug discontinuation was similar between treatment groups. No patients discontinued the study due to an AE. There were 2 AEs that led to study drug discontinuation in the prasugrel arm, but neither of these events was hemorrhagic or deemed by the investigator to be related to the study drug. The events were pain not otherwise specified and bronchitis. There were no clinically significant differences in the routine laboratory values (complete blood count, chemistry panels, and liver function tests) with serial follow-up (data not shown).

**Table 2:** Summary of adverse events

	**Prasugrel**	**Placebo**
	**(n = 41)**	**(n = 19)**
**Any hemorrhagic event, n (%)**		
**Required medical attention**	0	0
**Treatment-emergent**	8 (19.5)	1 (5.3)
**Possibly related to study drug**	6 (14.6)	1 (5.3)
**Any non-hemorrhagic event, n (%)**		
**Serious**	8 (19.5)	4 (21.1)
**Study drug discontinuation**	2 (4.9)	0
**Treatment-emergent**	31 (75.6)	17 (89.5)
**Possibly related to study drug**	2 (4.9)	0
**Any event, n (%)**		
**Serious**	8 (19.5)	4 (21.1)
**Study drug discontinuation**	2 (4.9)	0
**Treatment emergent**	34 (82.9)	17 (89.5)
**Possibly related to study drug**	8 (19.5)	1 (5.3)

**Table 3:** Hemorrhagic adverse events

**Treatment**	**Patient**	**Preferred term**	**Severity**	**Possibly related to study drug**
**Placebo**	**1**	Hematochezia	Mild	
		Gingival bleeding	Moderate	Yes
		Hemorrhoids	Mild	
**Prasugrel**	**2**	Menorrhagia	Moderate	Yes
	**3**	Menorrhagia	Mild	Yes
		Ecchymosis	Mild	
	**4**	Epistaxis	Mild	Yes
	**5**	Contusion	Mild	Yes
	**6**	Epistaxis	Mild	
	**7**	Gingival bleeding	Mild	Yes
	**8**	Gingival bleeding	Mild	Yes
	**9**	Hematochezia	Mild	

**Figure 3:**
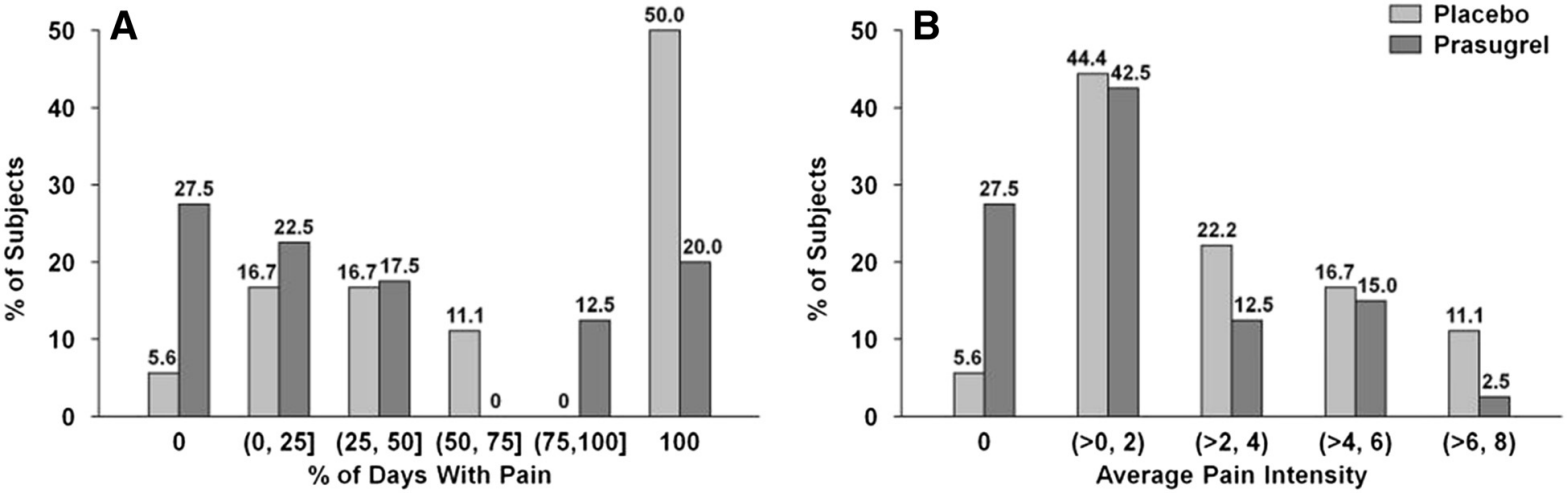
**Patient-reported days with pain and pain intensity. A**. Proportion of patients reporting pain on 0, >0 to 25, >25 to 50, >50 to 75, >75 to <100, or 100% of study days. **B**. Proportion of patients with average pain intensity of 0, >0 to 2, >2 to 4, >4 to 6, or >6 to 8. Prasugrel = black bars; placebo = grey bars

The efficacy endpoints in this study were the rate and intensity of pain as recorded by daily self-administered pain diaries. The mean completion percentage for diaries (number of entries/total possible days) was 95.7% for the prasugrel arm and 92.6% for the placebo arm. There were numerical decreases in proportion of days with pain and pain intensity in the prasugrel treated patients: a 21% relative reduction (least-squares [LS] mean: 42.2% vs. 53.5%) in pain rate and a relative 25% reduction (LS mean: 1.8 vs. 2.4) in pain intensity. However, these differences did not reach statistical significance. An alternative analysis showed that a greater proportion of patients in the prasugrel arm (50%) reported no pain throughout the study (27.5% of patients with a percentage of days with pain = 0) or infrequent pain (22.5% of patients with ≤ 25% of days with pain) versus 22.3% of placebo-treated patients having either no pain (5.6% of patients with a percentage of days with pain = 0) or infrequent pain (16.7% of patients with ≤ 25% of days with pain) (Figure 3A). Nine of 40 (22.5%) of prasugrel patients had painful episodes during the study period versus 7/19 (36.8%) in the placebo group. Again, this was not statistically different.

**Figure 4:**
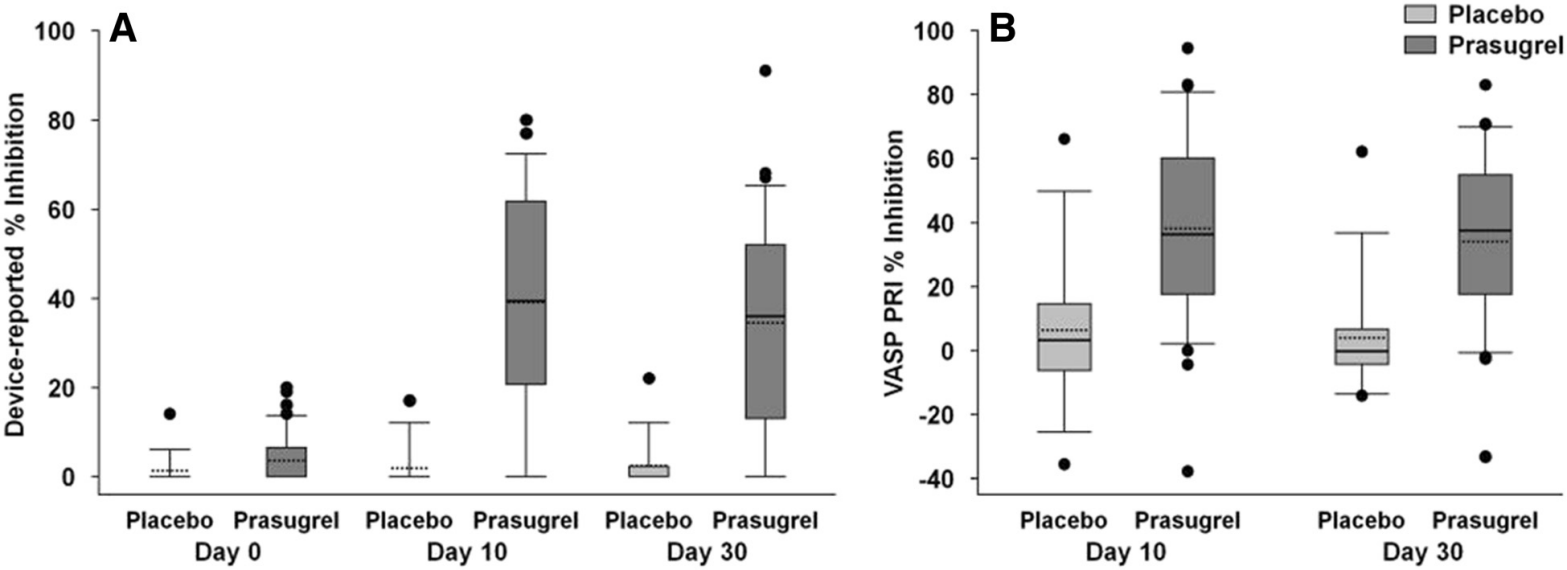
**Pharmacodynamic effects of prasugrel on platelet function. A**. Platelet Inhibition: VerifyNow® P2Y12. **B**. VASP platelet reactivity index. The bottom and top of the box are the 25th and 75th percentile, the solid line in the box is median and the dotted line is mean, the ends of the whiskers are 10th and 90th percentile

The point-of-care VerifyNow® P2Y12 device and VASP PRI were used to assess prasugrel’s pharmacodynamic effect on platelets. Figure 4 shows that by both measures, 5 mg prasugrel resulted in statistically significant greater platelet inhibition compared to placebo (*P* < .001) at both day 10 and day 30. About 20% of patients had <20% inhibition, which is consistent with the rate seen with the 5-mg maintenance dose in previous studies [24-26]. Because the proportion of patients did not fulfill the pre-specified criterion for escalation, the dose remained at 5 mg for the duration of the study.

**Figure 5:**
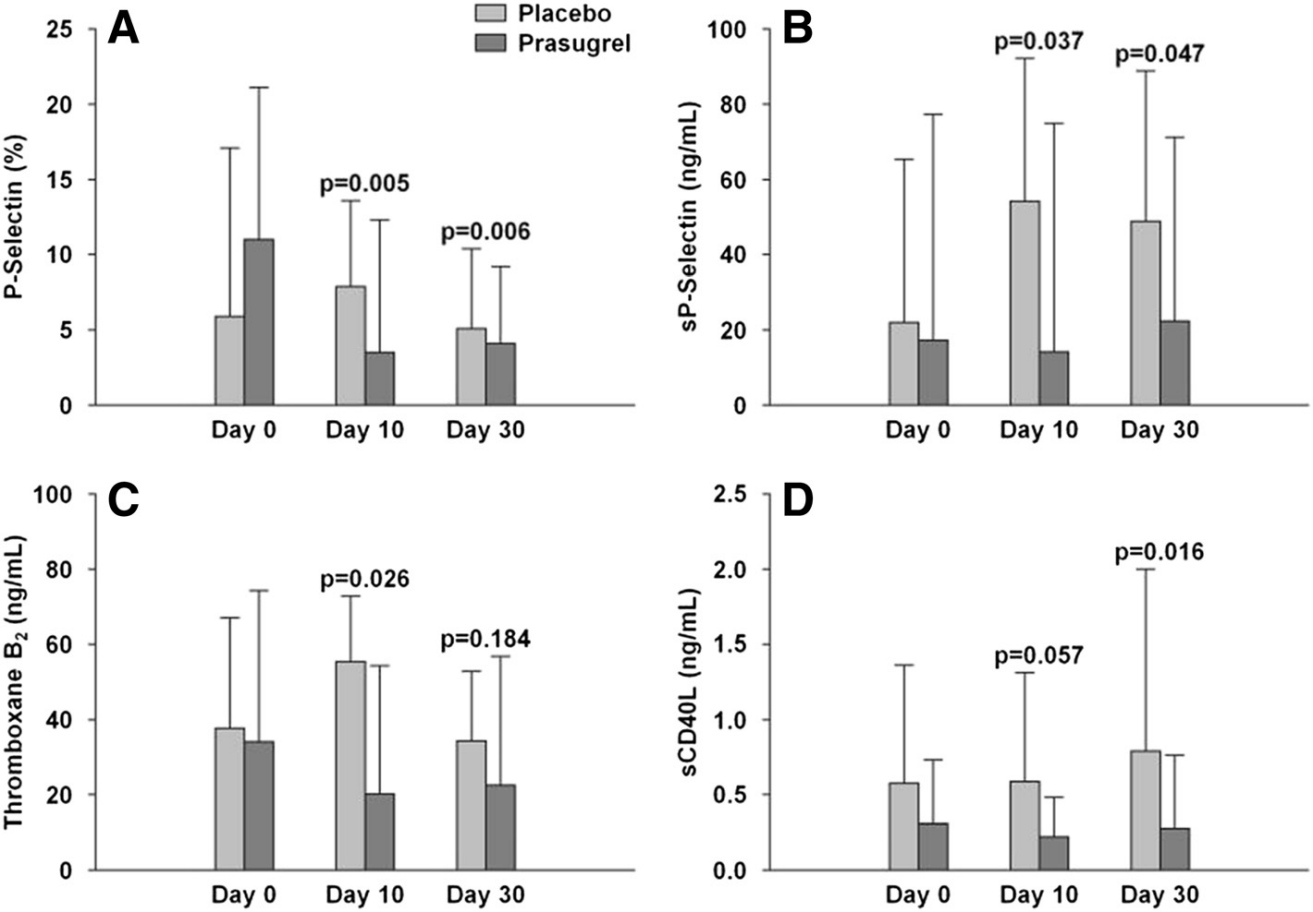
**Effect of prasugrel vs. placebo on biomarkers of disease-related platelet activation. A**. Percent of platelets positive for platelet surface P-selectin, **B**. Plasma soluble P-selectin, **C**. Serum TXB2, **D**. plasma soluble CD40L. Prasugrel = black bars; placebo = grey bars. Results are mean ± SD

Numerous studies have shown that platelets are activated in SCD patients [10,12] and that markers of platelet activation are associated with complications [13,14]. The effects of prasugrel on platelet activation were measured with both cellular (platelet P-selectin) and soluble biomarkers (TXB2, soluble CD40L, and soluble P-selectin). There were statistically significant decreases in platelet P-selectin and soluble P-selectin at both days 10 and 30 in the prasugrel arm compared to placebo. The decreases also reached statistical significance for TXB2 at day 10 and for soluble CD40L at day 30 in the prasugrel arm compared to placebo (Figure 5). Therefore, prasugrel was shown not only to inhibit platelet function, but also to decrease platelet activation in patients with SCD.

## Discussion

In this randomized Phase 2 study of prasugrel in adult patients with various genotypes of SCD in steady state, 30 days of treatment was not associated with hemorrhagic events that required immediate medical attention. As expected for a platelet function inhibitor, there were more hemorrhagic AEs with prasugrel; however, the majority was mild and none resulted in serious adverse events or adverse events leading to study drug or study discontinuation. However, it bears emphasis that exclusion criteria were conservative, enrolled subjects were at low risk for hemorrhage and this pilot study was of short duration.

Evidence of platelet inhibition was seen amongst prasugrel-treated patients with SCD. Prasugrel treatment resulted in approximately 33% to 40% platelet inhibition as measured by both VerifyNow® P2Y12 and VASP, suggesting that patients with SCD achieved a substantial pharmacodynamic response with prasugrel treatment similar to that seen in previous studies in patients with cardiovascular disease. Despite this effect, it was still possible that the marked hemostatic and inflammatory milieu in SCD patients might have overcome potential inhibition of platelet activation by prasugrel via other platelet activation pathways, such as thrombin generation. However, the decreased levels in cellular and soluble markers of platelet activation observed in the prasugrel-treated group show this agent is able to overcome this platelet pro-stimulatory environment and attenuate *in vivo* platelet activation in SCD without producing serious bleeding.

Perhaps more provocative is the suggestion that prasugrel may be efficacious in reducing pain. The traditional endpoint for therapeutic efficacy in SCD is the occurrence of acute painful episodes (pain that requires an unscheduled visit to a medical facility for treatment). However, paradigm shifting work by Smith et al. [27,28]. has shown that chronic pain is common in patients with SCD. Therefore, reductions in pain rate (the proportion of days with pain) and pain intensity over time are important endpoints for SCD therapy to assess efficacy in treating the different types of pain problems experienced in this patient population. A numerical decrease in pain rate and intensity compared with placebo, as reported by daily patient diaries, was seen in this study. There was also a numerical decrease in acute pain episodes compared with placebo. Neither of these decreases reached statistical significance, and this pilot study was not designed to provide definitive conclusions regarding pain. However, the number of patients who reported days with zero pain was different. This latter finding was intriguing, as it is a measurement of reduction of both acute pain episodes and chronic pain and was seen after only 30 days of treatment, a more rapid time to response than seen with hydroxyurea [1].

It is important to compare and contrast the results of this study to the other published studies of platelet inhibitors in SCD. Osamo et al. examined the effect of aspirin on red cell survival in patients with SCD [15]. Fifty patients aged 11–20 years with HbSS were assigned to 1200 mg daily of soluble aspirin in divided doses for 6 weeks; another 50 were assigned to usual care only. Hemoglobin and oxygen saturation levels increased in the treated group, and red cell survival increased in the 3 patients in whom it was studied. They also demonstrated a shift in the electrophoretic mobility of HbS in the aspirin-treated patients indicating chemical modification of HbS. Pain was not formally assessed in this study, and no serious hemorrhagic events were reported.

Greenberg et al. studied somewhat lower doses of aspirin (3–6 mg/kg) for 21 months in 49 children with HbSS, HbSC, or HbSO-Arab in a double-blind cross-over study to prevent acute pain crises [29]. Ninety-four patients were originally enrolled but analysis was per protocol: only 49 that were determined to be at least 50% adherent with the study drug were included in the analysis cohort. There was no difference in the number of painful episodes, number of total days in pain, duration of pain crisis, or pain severity during crisis between the aspirin- and placebo-treated periods. Interestingly, there was a marked decrease in the number of pain crises after the first 6 months on-study, irrespective of the treatment assignment.

Chaplin and colleagues added the phosphodiesterase inhibitor dipyridamole to aspirin as prophylaxis for acute pain crisis [30]. This study included only 3 patients treated with aspirin 650 mg PO and dipyridamole 50 mg PO both twice daily and compared the frequency and severity of pain for the 2 years on therapy to the 2 years not on therapy. The severity of pain appeared to be less while on therapy, and the total number of hospitalizations for pain decreased as well.

Previous studies have tested the effect of the first generation thienopyridine ticlopidine in SCD patients. Semple and colleagues assessed platelet survival in 9 patients with SCD using radiolabeled platelets and platelet activation by measuring plasma levels of platelet release products [17]. Patients were randomized to placebo or ticlopidine 250 mg PO twice daily for 28 days. Ticlopidine did not prolong platelet survival but did decrease markers of platelet activation, as was seen in our study. They noted an approximate 40% reduction in collagen and ADP-induced maximal platelet aggregation. The inhibition of platelet function and relative reduction in markers of platelet activation is similar, in degree, to that seen in the present study using different assays. One patient had a painful episode while on active drug, but clearly this study was not powered to determine a difference in pain. Adverse events were not reported.

Cabannes et al. studied the efficacy of ticlopidine in the prevention of acute pain crisis in 140 patients in Africa [6]. Patients were randomized 1:1 between ticlopidine 500 mg to 750 mg daily (dependent on body weight) or placebo for 6 months of therapy. Although the precise definitions of crisis, crisis duration, and crisis severity cannot be discerned from the report, all three of these parameters were statistically significantly decreased in the ticlopidine arm compared with the placebo arm. No platelet activation or survival markers were assessed.

Our study showed similar effects on platelet activation to those demonstrated with ticlopidine, confirming its platelet suppressive effect in this population of patients without provoking clinically serious bleeding. It is important to note that the current Phase 2 study was not powered to find a difference in acute painful episodes. Nonetheless, there was a trend toward decreased number of days with pain. Treatment with prasugrel compared with placebo was associated with numerical decreases in pain rate and pain intensity, as reported in daily patient diaries, and a numerical decrease in pain events related to SCD that required medical attention, as assessed by the study investigator. In adjusted analyses, prasugrel was associated with a 21% relative reduction in the percentage of days with pain and a 25% relative reduction in pain intensity compared with placebo.

Although it is clear from the patient pain diaries that the majority of patients had pain at some point during the study and many of the patients randomized to placebo experienced chronic pain, relatively few patients sought medical attention for their pain (27%). From the case report forms, 22.5% of prasugrel-treated patients sought medical attention for a pain event versus 36.8% of placebo-treated patients. These provide a signal that prasugrel may be efficacious in reducing the frequency and severity of VOC in patients with SCD.

## Conclusion

In summary, the results of this randomized, double-blind, Phase 2 study showed that prasugrel inhibited platelet function, decreased biomarkers of platelet activation, and showed a trend toward decreased pain by several measures without serious hemorrhagic adverse events. However, these results must be interpreted with caution, as measures of pain were not primary outcomes and the duration of this study (30 days) was short. A larger study with a sample size appropriately powered to determine effects on clinically relevant endpoints and provide longer safety analysis is needed to confirm and extend these findings.

## Competing interests

CZ, LEH, KJW, JSR, and JAJ, Eli Lilly and Company Employment and Equity Ownership. TW, LK, LEM, JJS, Daiichi Sankyo Co., Ltd. and Eli Lilly and Company Research Funding. CEN, Daiichi Sankyo, Inc. Employment and Equity Ownership. AK, Research funding from Novartis, Celgene, Hemaquest, Daiichi Sankyo Co., Ltd., and Eli Lilly and Company. KIA, Adventrx Consultancy, Daiichi Sankyo Co., Ltd. and Eli Lilly and Company Research Funding. ALF, PhD Research funding: Daiichi Sankyo Co., Ltd. and Eli Lilly and Company, GLSynthesis, Glycomimetics; Consultant: Eli Lilly and Company. CLK, DS, EN, no financial relationship(s) to disclose.

## Authors’ contributions

TW, participated in the design of the study, chaired the study, performed the research, and wrote the manuscript. ALF III performed research (assayed selected biomarkers); contributed to manuscript EN, performed research (contributed patients to the study), contributed to manuscript DS, FRCPC, MSc, performed research (contributed patients to the study), contributed to manuscript. LK, performed research (contributed patients to the study), contributed to manuscript. AK, performed research (contributed patients to the study), contributed to manuscript KA, performed research (contributed patients to the study), contributed to manuscript. CZ MS, participated in the design of the study, analysis and interpretation of the data, contributed to manuscript and performed the statistical analyses. LEH, participated in the design of the study, analysis and interpretation of the data, contributed to manuscript. CEN, participated in the design of the study, contributed to manuscript. JAJ, participated in the design of the study, analysis and interpretation of the data, contributed to manuscript. KJW, participated in the design of the study, analysis and interpretation of the data, contributed to manuscript. JSR, participated in the design of the study, contributed to manuscript. CLK, performed research (contributed patients to the study), assisted with revisions of the manuscript. LEM, Performed research (contributed patients to the study), contributed to manuscript. JJS Performed research (contributed patients to the study), contributed to manuscript. All authors read and approved the final manuscript.
